# Impact of sanitary and phytosanitary measures on agri-products quality upgrading and environmental protection

**DOI:** 10.1371/journal.pone.0297787

**Published:** 2024-04-05

**Authors:** Xuemei Liu, Haojun Qiu

**Affiliations:** 1 Alibaba Business School, Hangzhou Normal University, Hangzhou, China; 2 Hangzhou International Urbanology Research Center and Center for Zhejiang Urban Governance Studies, Hangzhou, China; National Technical University of Athens: Ethniko Metsobio Polytechneio, GREECE

## Abstract

Protecting human, animal, and plant life or health from additives, toxins, and contaminants in agri-products and promoting green free trade are the main components of Sanitary and Phytosanitary (SPS) measures. However, the SPS measures are heterogeneous. This study examines the impact of SPS measures on the measured export quality and discusses their influence on the environmental protection of the exporting country. International heterogeneous measures do not necessarily promote quality upgrading but greatly increase transaction costs. By contrast, China’s agri-product’ quality upgrading and environmental pollution are in sharp contrast. Based on a heterogeneous firm-trade model, this study obtains three hypothetical propositions and conducts empirical regressions using the Tobit method. This study finds that heterogeneous SPS measures hinder quality upgrading because firms present a different quality upgrading trend, which in turn impedes the environmental protection of the exporting country; the quality upgrading made by diversified SOEs is higher than that of foreign firms and private firms; the quality upgrading made by general firms is higher than that of processing firms; and protective SPS measures have a stronger negative effect on quality upgrading and environmental protection.

## Introduction

Many environmentalists argue that the WTO (World Trade Organization) needs to be “greened” and WTO rules are insensitive to environmental objectives (Charnovitz, 2007; Gentile, 2009; Diebold, 2010) [1–3]. Since WTO policy favors free trade and environmental policy calls for measures (tariffs, Sanitary and Phytosanitary measures, Technical Barriers to Trade, etc.) that may restrict trade, the two perspectives sometimes clash(Neumayer,2000;Zelli,2017) [4, 5]. Countries use trade measures to address the environmental degradation that occurs beyond their borders. These measures can be considered product-related pollution measures. Indeed, as product measures have improved, quality improvement has become an important way to avoid environmental pollution and maintain green free trade(Sadat & Alom, 2016) [6].

The international agri-product market operates under multilateral rules. On the one hand, the WTO encourages members to comply with the international standards promulgated by the Codex Alimentations Commission (CAC) (Randall et al.,1998; Wieck & Grant, 2021) [7, 8]; on the other hand, to satisfy the quality preferences of customers from different importing countries and to ensure high-quality green free trade, the WTO also recognizes SPS measures formulated by various countries (Rigod, 2013) [9] Meanwhile, product-caused pollution broadly includes harm to human, animal, or plant life, as well as harm to the environment, which are the main components of the SPS measures that affect green free trade (Liu et al., 2022) [10].

As a “quality threshold”, SPS measures are growing rapidly and are being implemented in an increasingly heterogeneous manner (e.g., diversification, protection and inconsistent) [11]. For example, based on the database of the Committee on Agriculture and Rural Development of the European Parliament, the statistical index of MRLs (Minimum and Maximum Residue Levels) shows that (1) in terms of national MRLs, Argentina, Australia, Mexico, and the United States are more stringent (≥ 1) than the EU, while Japan, South Africa, Israel, Ukraine, and other countries (0.8–1.037) are less stringent than the EU. However, China and other countries (0.72–0.78) are just meeting the CAC measures, and Canada and New Zealand are more lax. (2) Rice and wine have much higher product-level MRLs than wheat, oranges, potatoes, apples, etc. Therefore, the heterogeneity of SPS measures is a universal phenomenon in global agri-product trade markets and has seriously impacted international agricultural trade.

Heterogeneous SPS measures do not necessarily promote the improvement of product quality and environmental protection, but greatly increase transaction costs [12, 13], particularly firms’ export costs from developing countries. However, China is only a large producer and not a country with strict measures. Faced with the protective pressure of heterogeneous international SPS measures and the structural reform of the domestic agricultural supply side, Chinese export firms usually have two choices: horizontal market diversification (market structure adjustment) and vertical quality upgrading (product structure improvement).

On the one hand, horizontal export market diversification can meet the different quality preferences of consumers, avoid the compliance costs of rising measures in the original markets, and maintain the stability of market profitability [14]; On the other hand, horizontal market diversification creates conditions for firms to evade high measures and will have a negative impact on vertical product quality upgrading. Hence, this may cause firms to lock into low-standard markets in the short run and fall into a quality-upgrading dilemma in the long run [15].

Therefore, studying the impacts of heterogeneous SPS measures is of great practical significance. Numerous studies have thoroughly investigated SPS measures and quality upgrading. This study primarily focuses on two types of literature: The first relates to SPS measures. Scholars have focused on the positive and negative effects of SPS on trade flows [16], the heterogeneous effect of SPS on quality upgrading at the national level [17], and the influence mechanism of SPS measures when integrated into product diversification [18]. In addition, some scholars have considered the different quality preferences of consumers [19] and used per capita income as a proxy index for quality preferences [20]. These results are still far from explaining the effect of heterogeneous SPS measures, especially when the selection of export firms in the multi-destination trade network is ignored.

The second type of literature relates to the multi-destination trade model. Scholars have mostly focused on the productivity of exporting firms [21–23], export path dependence [24], effect of the exchange rate [25], effect of anti-dumping [26], and trade flows. They did not include trade costs (e.g., SPS compliance costs and export market-switching costs). However, if the production costs are higher than the marginal cost of the destination country, the exporting firms must withdraw from the original export market [21]. However, Chinese trade facts indicate that exporting firms face global markets, and that trade costs differ in different destinations. An increase in the original export destination’s trade cost does not necessarily cause firms to withdraw, and they can find alternative destinations with lower trade costs to switch export markets [27].

The third type of literature relates to trade environment. Barcelo [28] discussed trade–environment conflicts over product standards that protect the environment of an importing country. Ekins et al. [29] suggested ways to facilitate trade that adequately protects the environment, sustainability, and other social values. Cole and Elliott [30] examined whether compositional changes in pollution resulting from trade liberalization are due to differences in capital–labor endowments and/or environmental regulations. Copeland and Taylor [31] critically reviewed both theory and empirical evidence on issues such as the environmental Kuznets curve, the pollution haven hypothesis, and the impact of environmental policy differences on trade and investment flows. Peters and Hertwich [32] used three complementary approaches to examine the Norwegian economy’s production network, which led to domestic and international environmental impacts. Anderson [33] reviewed the role of food policy in changing the environment and proposed alternatives to current policies that could better achieve national societal goals while benefiting the rest of the world in terms of reducing natural resource and environmental pressures and reducing national and global poverty, food and nutrition insecurity, and inequalities in income, wealth, and health. Udeagha and Breitenbach [34] revisited the dynamic relationship between trade openness and carbon dioxide (CO2) emissions for Southern African Development Community (SADC) member countries and found that increased trade openness improves environmental quality. However, few scholars have focused on the impact of SPS standards on trade and the environment.

In this study, based on the firm_level data(2000–2015) and the Tobit method, we try to examine the impact of heterogeneous SPS measures on firms’ export choices in the short run and the impact on environmental protection in the long run. It is expected to study the impact of heterogeneous standards on environmental protection from the perspective of SPS measures, and how to influence the export choices of firms, which play a major role in global trade. The remainder of this paper is organized as follows: “Section 2: Stylized facts” presents stylized facts on Chinese agri-product exports. “Section 3: Theory hypothetical propositions” presents the theoretical and hypothetical propositions. “Section 4: Empirical framework and data” presents the empirical model and describes the data. “Section 5: Results” reports and explains the estimation results. Finally, “Section 6: Discussion on quality upgrading and environmental protection” discusses the main findings and provides policy recommendations. Finally, “Section 7: Conclusion” concludes the paper.

## Stylized facts

### Agri-product quality and environmental pollution in China

**Fig 1:**
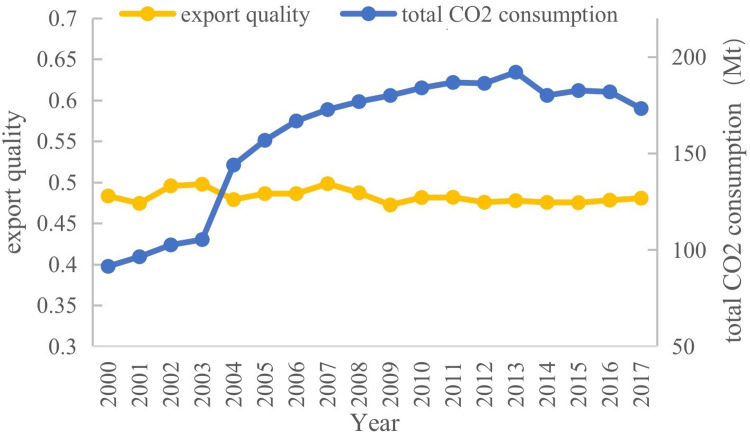
Agri-food quality export to U.S. and CO2 consumption in China (2000–2017). NOTE: Authors’ estimates using data from Schott’s personal website and the CEADs.

Based on the method of Khandelwal et al. (2013), we measure the product quality at HS10 code using the transportation cost at HS10 code as the instrumental variable (as shown in S1 Appendix). Due to the huge differences in trade barriers and income levels in different destinations, we can only compare the same category of products from the same importing country, which faces the same social preferences, per capita income, consumption habits, and market rules. Therefore, this study considers the United States, which is the second-largest importer of agriproducts and the second-largest export destination in China, as the importing country. Considering the endogeneity of price and quality at the product level, we use transportation cost as the instrumental variable, where the data are obtained from the Schott website (https://sompks4.github.io/sub_data.html), which provides trade data between the United States and the world. Therefore, based on the DSM quality measurement model [35], we conducted 5,088 regressions to estimate the product quality of 233 countries exporting to the US during 2000–2017. We obtained the quality levels of China’s agri-products exported to the U.S. (yellow line in Fig 1) and China’s total CO2 consumption (blue line in Fig 1). The comparison shows that the quality of China’s agri-products exported to the US is generally on a gentle upward trend, whereas CO2 consumption shows a rapid upward trend. The trends in quality improvement and pollution are in sharp contrast.

### Quality distribution on firm level

**Fig 2:**
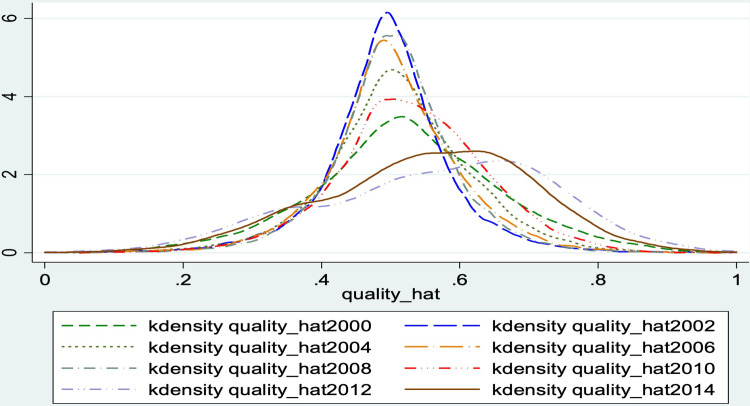
The quality level of China’s agricultural export firms (2000–2015). NOTE: Authors’ estimates are based on the General Administration of Customs of the People’s Republic of China (GACC). Owing to the lack of transportation costs at the firm level, we use the national exchange rate as an instrumental variable to measure Chinese firm-level export quality from 2000 to 2015.

In terms of exporting firms’ performance (Fig 2), the quality core density distribution of China’s agricultural firms between 2000 and 2015 was mainly concentrated in the middle. An increase in the number of firms reduces overall average export quality. This indicates that the uneven distribution and heterogeneous upgrading of firms have limited the overall upgrading of export quality.

### Regional environmental pollution

**Fig 3:**
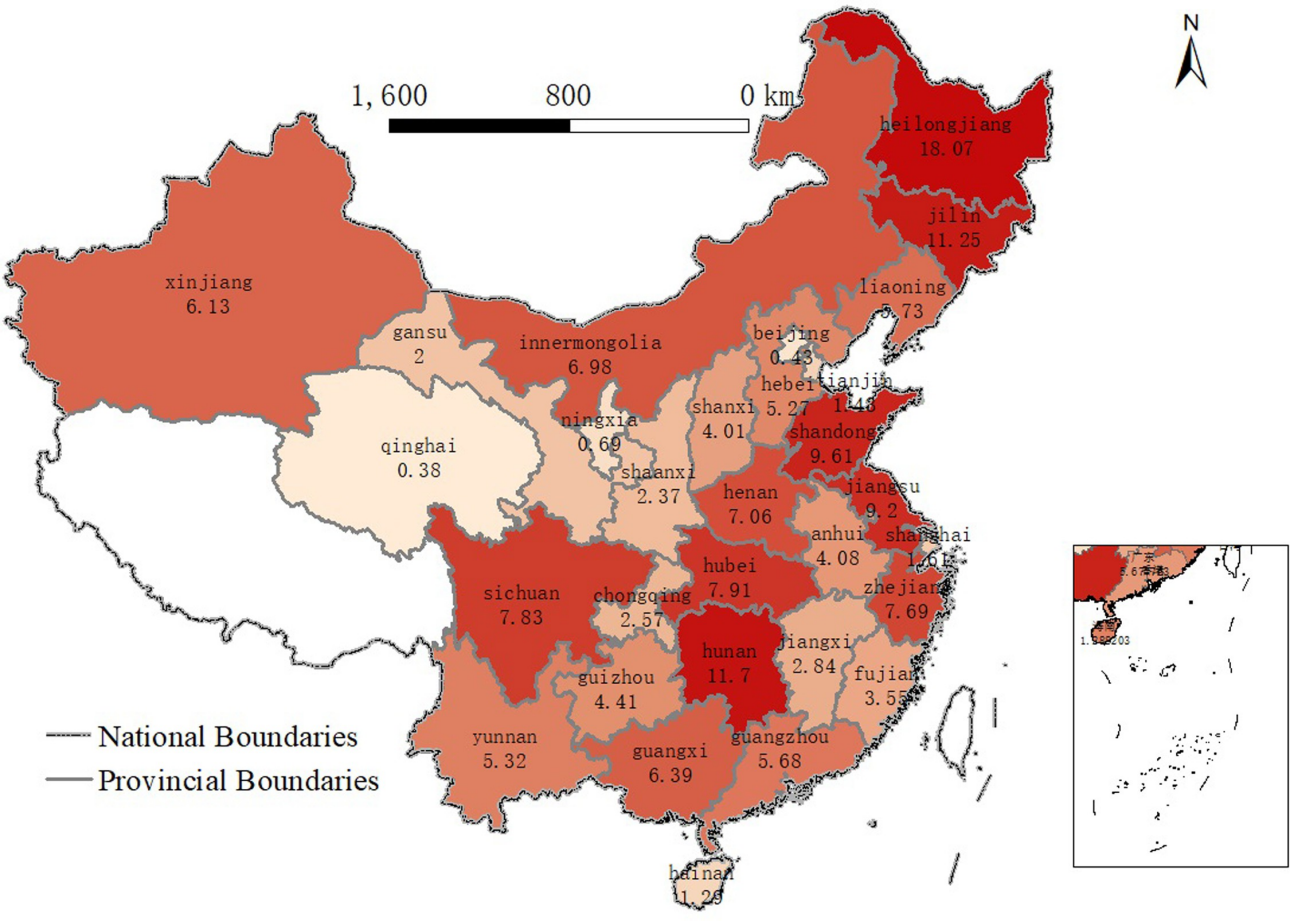
CO2 emitted by agri-food industries in China’s provinces in 2019 in 2019 (Unit: Mt CO2). NOTE: Data are from CEADs. Agri-food-related industries include farming, forestry, animal husbandry, fishery, water conservation, food processing, food production, and beverage production. Drawn through ArcMap 10.8. Esri reserves the right to grant permission for any other use of the Image.

In Fig 3, we found that (1) the amount of carbon emissions varied from province to province. (2) Important agricultural provinces in China are large carbon emitters from agricultural production, such as Heilongjiang (18.07), Jilin (11.25), Shandong (9.61), Jiangsu (9.2), and Sichuan (7.83). Simultaneously, there are also some large agricultural provinces that do not emit large amounts of carbon from agricultural production, such as Inner Mongolia (6.89) and Hebei (5.27), but the quality of agri-products in these provinces may be negatively affected by their large industry-wide carbon emissions, such as Inner Mongolia (794.28) and Hebei (914.21); the environmental factors (e.g., air, soil, and water) that farmers or agribusinesses use to produce agri-products differ. Environmental pollution can significantly reduce the quality (taste, freshness, and color) of agri-food products. Therefore, China started to adopt measures on agricultural non-point source pollution (e.g., put forward the goal and task of “one control, two reduction, and three basic” in 2015, launched the “five major” measures for agricultural green development in 2017. These measures implemented a series of actions, such as controlling the total amount of agricultural water consumption and agricultural water pollution; reducing the use of chemical fertilizers and pesticides; promoting the recycling of livestock and poultry waste, agricultural film, and crop straw; using organic fertilizers; and protecting aquatic organisms in key areas such as the Yangtze River.

### The heterogeneity of international SPS standards

In terms of total SPS measures (2000–2015), 123 WTO Members initiated 17915 SPS notifications. The top five initiators were the United States (1808), the European Union (1517), the Philippines (1326), Japan (1268), and Brazil (1037). According to statistics from the WTO SPS database (Table 1), the annual change in the number of SPS notifications increased almost five-fold from 2000 (363) to 2015 (1814), while the number of countries implementing SPS measures also increased by 44% from 2000 (38) to 2015 (55). These results indicate not only an overall upward trend in the number of SPS measures but also an increasing number of implementing countries.

**Table 1:** Descriptive statistics of SPS measures from 2000 to 2015 (Unit: Case).

Year	SPS notifications	countries/regions
**2000**	363	38
**2001**	882	50
**2002**	839	43
**2003**	840	43
**2004**	1070	46
**2005**	803	48
**2006**	1076	47
**2007**	1011	39
**2008**	1263	47
**2009**	1157	45
**2010**	1120	46
**2011**	1185	49
**2012**	1088	48
**2013**	1580	48
**2014**	1824	53
**2015**	1814	55

NOTE: data from WTO-SPS

**Fig 4:**
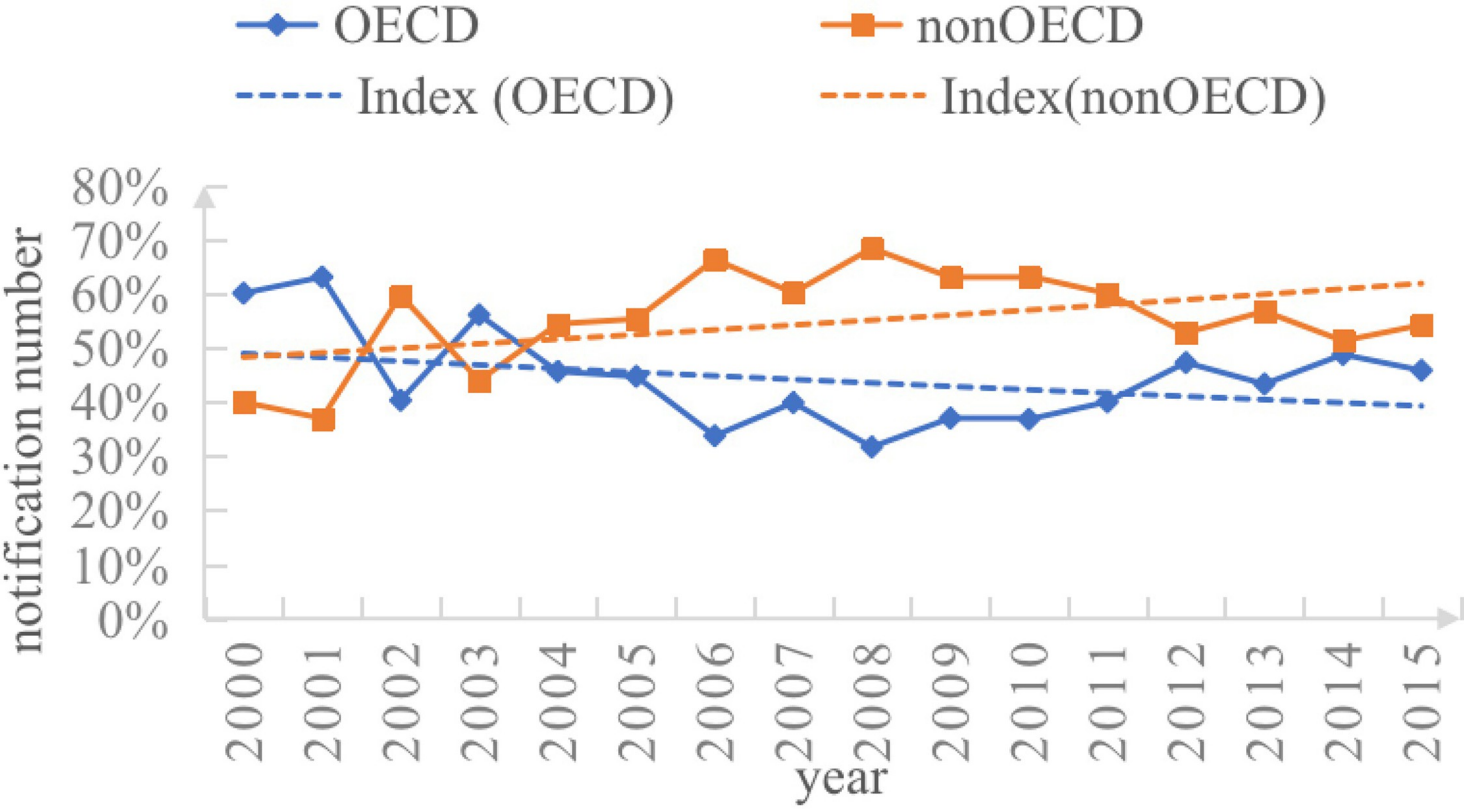
SPS notification by country (2000–2015). NOTE: data from WTO-SPS.

Fig 4 shows the share of SPS notifications in OECD and non-OECD countries. There were significant structural changes in the global growth of SPS notifications. From 2000 to 2015, non-OECD countries (10,087) accounted for 56.30% of the total number of notifications, which is more than the number of SPS notifications initiated by OECD countries (7,828), which accounted for 43.69%. It is important to note that although the number of SPS notifications from non-OECD countries is increasing, the inhibiting effect of the SPS measures of OECD countries still occupies the main position because of the heterogeneous measure levels between OECD and non-OECD countries.

### Theory hypothetical propositions

Based on Antoniades (2015), the optimal levels of product quality (${\bar{z}}_{lh}^{h}$) for foreign market are given below

$${\bar{z}}_{lh}^{h}=\lambda_{lh}^{h}\left[\frac{1}{k+1}\right]c_{D}^{l}$$ (1)

Where $\lambda_{lh}^{h}$ are the scopes for quality differentiation in the domestic and foreign markets and $c_{D}^{I}$ is the cost cutoff in the free trade economy. k is from the cost Parto distribution given by $G(c)={\left(\frac{c}{c_{m}}\right)}^{k},c\in \left[0,C_{m}\right].$ Obviously, under the bilateral trade model, export costs $\left(C_{D}^{I}\right)$ affect the quality level of country l exports to country h. However, under the multilateral trade model, the export costs are also affected by the different degrees of quality standard(s) in different destinations. From the analysis of the heterogeneity of the SPS standards in section 2.4, it is known that the standard differentiation among N countries is significant. Therefore, under the multilateral trade model, the export quality(${\bar{z}}_{lh}^{h}$) formula of country l exports to country h will be as follows:

$${\bar{z}}_{lh}^{h}=\lambda_{lh}^{h}\left[\frac{1}{k+1}\right]c_{D}^{l}(s)$$ (2)

To further analyze the impact of market diversification on export quality, it is essential to combine the different compliance costs directly caused by s and the transfer costs indirectly caused by s, which is the cost when firms withdraw from a high-standard $\left(\bar{S}\right)$ destination and export to a low-standard $\left(\underline{S}\right)$ destination. Besides, it is also necessary to combine the firms’ quality upgrading ability and we divide the firms into the leaders and the laggards according to the ABGHP model [36].

First, from a vertical perspective, this section analyzes the compliance costs caused by raising SPS measures from the same market. Although firms face the same quality measures, the distribution of export costs of laggards and leaders is different because of their different technical upgrading capabilities. Whether firms accept compliance costs and upgrade quality depends on the return on compliance costs induced by upgrading equipment, improving inspection and quarantine technology, etc. Even though compliance costs are more challenging for laggards, in order to earn more “Schumpeter rent,” the laggards are more affected by “learning-by-doing” and have a stronger impetus (higher return on compliance costs) to upgrade quality than the leaders. Therefore, the first hypothesis of this study is that the higher the return on compliance costs of SPS measures, the higher the quality. Under the “learning-by-doing” effect, laggards make more quality improvements than leaders.

Second, from the horizontal perspective, this section considers different destinations because export costs differ across destinations and include compliance costs (vertical perspective) and switching costs (horizontal perspective). The switching costs are mainly due to trade resistance when export firms try to enter a new market (e.g., standard thresholds, degree of trade liberalization, and trade distance). Faced with the heterogeneous SPS measures of importing countries, exporting firms have two export options. (1) Firms that are more dependent on the original export route are more inclined to improve their quality of the original route by accepting the compliance costs of SPS measures. (2) Firms that are less dependent on their original export route are more inclined to switch their destinations to low-standard countries and are more likely to accept switching costs. Hence, exporting firms can be divided into undiversified and diversified, assuming that the degree of path dependence of the former is higher than that of the latter. Thus, this paper obtains the second hypothesis: because of the “export switching” effect of diversified firms and the “path dependence” effect of undiversified firms, undiversified firms are more inclined to accept the compliance costs and upgrade quality more than diversified firms.

Finally, from a two-dimensional perspective, considering the vertical differentiation of compliance costs and the horizontal differentiation of switching costs simultaneously, this part classifies firms into four types: Undiversified laggards are mainly encouraged by the positive effects of “path dependence” and “learning-by-doing;” undiversified leaders are lacking innovation impetus and are mainly affected by the positive effect of “path dependence;” diversified laggards are mainly affected by the positive effect of “learning-by-doing;” and the fourth type is the diversified leaders, which not only lack the impetus to upgrade, but are also affected by the negative effect of “export switching.” In addition, considering the effectiveness of the “path dependence” effect and the “learning-by-doing” effect, “path dependence” effect is mainly affected by the external factors on firm level (e.g., the national geopolitics, the degree of market competition of the new target market, and the export experience of the firm), while the “learning-by-doing” effect is mainly affected by the internal factors of the firm (e.g., worker training, machinery and equipment update.). However, it is difficult to compare them because they are influenced by different dimensions. Facing the international heterogeneous SPS measures, under the effect of “path dependence” and “learning-by-doing,” undiversified laggards will innovate and reduce using additives, toxins, and contaminants, thus protecting the environment. However, if diversified firms choose to switch to low-standard countries, or if the leaders lack the impetus to upgrade quality, it is negative for environmental protection.

**Fig 5:**
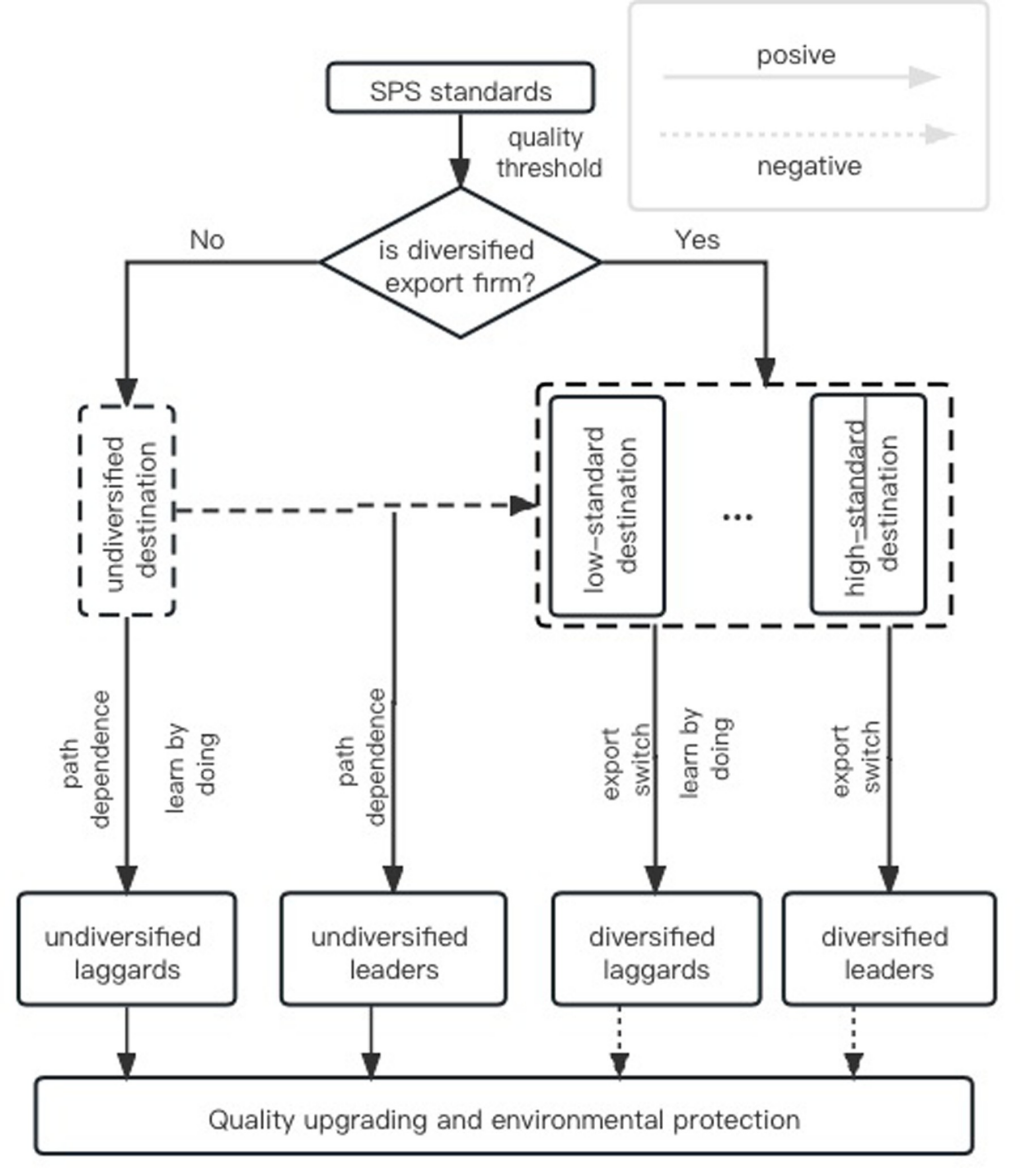
The track map of the SPS standard forces quality upgrading.

Therefore, this study obtains the third hypothetical proposition: international heterogeneity has a negative effect on environmental protection due to different firm export selections (as shown in Fig 5).

### Empirical framework and data

#### Empirical framework

To demonstrate the above 3 hypothetical propositions, this study uses firm-level GACC data from 2000 to 2015 and sets the following regression equation:

$${\text{Delt}}_{{\text{quality}}_{\text{eht}}}=\alpha_{\text{e}}+\alpha_{\text{h}}+\alpha_{\text{t}}+\beta_{1}{\text{SPS}}_{\text{eht-1}}+\beta_{2}{\text{FD}}_{\text{eht}}+\beta_{3}\;{\text{SPS}}_{\text{eht-1}}*{\text{FD}}_{\text{eht}}+\beta_{4}\;{\text{Adjusted}}_{{\text{price}}_{\text{eht}}}+\beta_{5}\;{\text{Tariff}}_{\text{eht}}+\varepsilon_{\text{eht}}$$ (3)

${\text{Delt}}_{{\text{quality}}_{\text{eht}}}$ represents quality upgrading on the firm level, which is equal to the quality change of firm e in country c export product h from year t to t-1. $\alpha_{e},\alpha_{h},\alpha_{t}$ represent firm fixed effect, HS2, code product level fixed effect and time fixed effect, respectively. SPS_eht-1_ represents the SPS notifications number of U.S. in year t-1, which is from the WTO-SPS database. FD_eht_ represents the front distance and it is measured by the quality level of one lag period (t-1). The closer it tends to 1, the more the firm is a quality leader; on the contrary, the closer it tends to 0, the more the firm is a laggards. As one of the control variables, ${\text{Adjusted}}_{{\text{price}}_{\text{eht}}}$ represents the quality-adjusted products price of different firms, which is mainly used to capture the marketing strategy and pricing strategies information covered by the price [37]; As one of the control variables, Tariff_eht_ represents the impact of tariff barriers on quality upgrading; *ε*_eht_ represents the disturbance term, including all other unobserved factors that affect the product quality. We performed regression analysis on undiversified and diversified export firms respectively to examine the heterogeneous impact of SPS measures on the quality upgrading of export firms.

### Data

We calculated product quality at the HS10 code level and combine it with the firm-HS8 code from the GACC database, which includes the year, trade value, quantity and transportation cost, firm code, firm ownership types (e.g., state-owned firms (SOEs), foreign firms, and private firms), and trade modes (e.g., general firms and processing firms). The agri-product sector mainly covers representative products (e.g., HS03, HS07, HS09, HS12, HS16, and HS20). Considering that the GACC database includes HS8 code product data, there is an overestimation of diversification in the statistics owing to the high-level product codes. Therefore, this study uses four destinations 4 (not one) to split the degree of diversification. By integrating the above data, 79,080 samples of China’s exports to the U.S. over the past 16 years were obtained. The descriptive statistics of the integrated data are presented in Table 2.

**Table 2:** Summary statistics.

Variables	Mean	Sd	Min	P50	Max
**Delt_quality**	0	0.37	-8.86	0	8.78
**SPS**	4.14	4.98	0	2	24
**FD**	6.27	2.42	0.09	5.91	10
**Adjusted price**	0.75	1.77	0	0.49	116.40
**Tariff**	6.46	4.26	0.26	8.36	12.85

## Results

### The effects of SPS standards on quality upgrading

Tobit and OLS (ordinary least squares) methods were used for the empirical regressions. We mainly find that (as shown in column 4 of Table 3): (1) SPS measures from the United States stimulate the ‘quality upgrading of Chinese agri-products. (2) Front distance has a significantly negative effect (-0.068) on both undiversified (-0.004) and diversified (-0.003) exporting firms. This proves Hypothesis 1, which states that increasing SPS measures forces laggards to improve quality more than leaders. (3) SPS measures stimulate undiversified firms (0.031) to upgrade their quality more than diversified firms (0.011). This proves Hypothesis 2, which states that SPS measures force undiversified firms to improve their quality more than diversified firms do. All these results confirm that because of the positive effect of “path dependence,” undiversified exporting firms have upgraded rapidly, and because of the negative effect of “export switching,” quality upgrading of diversified exporting firms is slower, which is mainly due to the fact that the higher the degree of market diversification, the richer the export experience. When firms have certain advantages in handling customs procedures, managing risks, researching new products, and entering new markets, they are more likely to switch destinations, especially in multilateral relationships with destinations that share common languages or colonial cultures [24].

**Table 3:** The effects of SPS standards on quality upgrading.

	Undiversified export firms			
	OLS-1	OLS-2	OLS-3	Tobit
**SPS**	0.004	0.046***	0.050***	0.031***
	(0.004)	(0.009)	(0.009)	(0.007)
**FD**	-1.227***	-1.146***	-1.161***	-0.068***
	(0.046)	(0.041)	(0.040)	(0.007)
SPS*FD		-0.006***	-0.008***	-0.004**
		(0.001)	(0.001)	(0.001)
**Adjusted**_**price**_		-0.135**	-0.129**	-0.184***
		(0.041)	(0.040)	(0.019)
**Tariff**			-1.223***	0.010**
			(0.266)	(0.003)
**Cons**	7.481***	7.071***	15.010***	0.446***
	(0.284)	(0.255)	(1.675)	(0.047)
**Obs**	4890	4890	4890	4890
**R** ^**2**^	0.674	0.692	0.703	
	**Diversified export firms**			
	OLS-1	OLS-2	OLS-3	Tobit
**SPS**	-0.008**	0.029**	0.036***	0.011*
	(0.003)	(0.009)	(0.010)	(0.005)
**FD**	-1.190***	-1.176***	-1.172***	-0.068***
	(0.023)	(0.023)	(0.023)	(0.004)
SPS*FD		-0.006***	-0.007***	-0.003***
		(0.001)	(0.001)	(0.001)
**Adjusted**_**price**_			-0.026**	-0.120***
			(0.009)	(0.009)
**Tariff**			-1.456***	0.015***
			(0.205)	(0.002)
**Cons**	7.684***	7.584***	16.642***	0.467***
	(0.148)	(0.152)	(1.258)	(0.025)
**Obs**	12747	12747	12747	12747
**R** ^**2**^	0.674	0.692	0.703	

NOTE: Values in brackets are standard deviations. ***, ** and * respectively mean statistical significance of 1%, 5% and 10%.

Considering the degree of market diversification and technological upgrading ability of firms, when faced with the pressure of international heterogeneous SPS measures, undiversified laggards (0.031) have the highest upgrade range, followed by undiversified leaders (0.027) and diversified laggards (0.011), while diversified leaders (0.007) have the lowest. The different quality-upgrading ranges indicate that international heterogeneous SPS measures distort agricultural firms from using additives, toxins, pesticides, and fertilizers, which is effective in improving productivity and not upgrading quality. This finding supports Hypothesis 3.

### Robustness test

Considering that intermediary firms find it easier to switch to a new destination when facing high-level SPS measures, they are less motivated to upgrade quality. Compared with land-intensive agri-products, labor-intensive agri-products can update the production process to meet the new requirements of SPS measures. To test for robustness, we examine the effect of SPS measures from the perspective of excluding trade intermediary firms, focusing on labor-intensive agri-products and PPML regression methods. For the definition of trade intermediaries, this paper adopts the identification method and defines as trade intermediaries those firms whose name contains the words “trading company,”““import and export,”““commerce and trade” [38]. Table 4 reports the regression results at different levels, which are consistent with the results in Table 3.

**Table 4:** The robustness test.

	Exclude the intermediary		Labor incentive products		PPML	
	Undiversified export firms	Diversified export firms	Undiversified export firms	Diversified export firms	Undiversified export firms	Diversified export firms
**SPS**	0.025**	0.002	0.007	0.015*	4.558***	3.924***
	-0.008	-0.005	-0.009	-0.006	(1.081)	(0.690)
**FD**	-0.071***	-0.073***	-0.136***	-0.106***	-0.169***	-0.172***
	-0.008	-0.004	-0.011	-0.006	(0.018)	(0.007)
SPS*FD	-0.004**	-0.002*	0.003	-0.003*	-0.908***	-1.257***
	-0.001	-0.001	-0.002	-0.001	(0.221)	(0.126)
**Adjusted**_**price**_	-0.187***	-0.120***	-0.191***	-0.167***	-28.788***	-27.845***
	-0.02	-0.009	-0.02	-0.012	(2.718)	(1.880)
**Tariff**	0.010**	0.018***	-0.010*	0.001	0.016*	0.028***
	-0.004	-0.002	-0.004	-0.003	(0.007)	(0.004)
**Cons**	0.482***	0.496***	0.852***	0.734***	-0.204*	-0.201***
	-0.051	-0.027	-0.063	-0.039	(0.098)	(0.047)
**Obs**	4277	11324	3196	8002	4890	12747

NOTE: values in brackets are standard deviations. ***, ** and * respectively mean statistical significance of 1%, 5% and 10%.

## Discussion on quality upgrading and environmental protection

### Heterogeneity across firms with different ownerships

Table 5 shows the ownership distribution of various diversified firms in China’s agri-product exports to the U.S. This extreme distribution shows that diversified private firms contribute significantly to the growth of agri-product exports. Therefore, the question arises: What is the export selection of firms with different ownerships? Table 6 presents the regression results for SOEs, private firms, and foreign firms. The comparative analysis shows that, (1) in general, regardless of the ownership of the firm, under the positive effect of SPS measures, the quality upgrading of undiversified firms is greater than that of diversified firms. (2) Among undiversified export firms, SPS measures significantly promote quality upgrading in private firms, whereas the positive effect on SOEs and foreign firms is not significant. Undiversified private firms rely mainly on their original export market because of limited financing and business scales. (3) Among diversified firms, the quality upgrading of SOEs is more obvious. SOEs have the advantages of capital, technology, and management, and can reduce export switching costs in response to SPS measures. (4) For both undiversified and diversified firms, the impact of SPS measures on the quality upgrading of foreign firms is not significant. Foreign firms have a special supply route; they can meet SPS measures easily and are transported to their original destination after processing abroad.

**Table 5:** Share of the export value of firms with different ownership in 2015 (Unit: %).

Ownership	Undiversified export firms		Diversified export firms	
	The proportion in undiversified export value (%)	the proportion in total export value (%)	the proportion in diversified export value (%)	the proportion in total value (%)
**State-owned firms**	17.01	1.33	82.99	6.48
**Private firms**	23.32	17.58	76.68	57.80
**Foreign firms**	21.69	3.64	78.31	13.16

Source: Estimated by the authors using GACC.

**Table 6:** The regression results for firms with different ownership.

	Undiversified export firms			Diversified export firms		
	State-owned	Foreign	Private	State-owned	Foreign	Private
**SPS**	0.013	0.023	0.044***	0.059***	-0.009	0.009
	(0.029)	(0.017)	(0.008)	(0.014)	(0.008)	(0.006)
**FD**	-0.083***	-0.083***	-0.049***	-0.039***	-0.085***	-0.077***
	(0.019)	(0.013)	(0.010)	(0.007)	(0.007)	(0.007)
SPS*FD	0.000	-0.002	-0.006***	-0.009***	0.001	-0.003**
	(0.005)	(0.003)	(0.001)	(0.002)	(0.001)	(0.001)
**Adjusted**_**price**_	-0.178***	-0.217***	-0.156***	-0.094***	-0.130***	-0.158***
	(0.045)	(0.033)	(0.025)	(0.014)	(0.016)	(0.017)
**Tariff**	0.019*	0.011	0.004	0.000	0.024***	0.022***
	(0.009)	(0.006)	(0.005)	(0.003)	(0.003)	(0.003)
**Cons**	0.478***	0.538***	0.357***	0.359***	0.535***	0.502***
	(0.124)	(0.083)	(0.063)	(0.049)	(0.042)	(0.045)
**Obs**	936	2019	1935	4780	4035	3932

NOTE: values in brackets are standard deviations. ***, ** and * respectively mean statistical significance of 1%, 5% and 10%. All the models were estimated using Tobit.

From the perspective of environmental protection, due to the limitations of firm-level data on environmental pollution, we can only discuss the different reflections of the SPS measures. On the one hand, SOEs have large-scale production, high capital, and technology to meet the SPS measures of many countries; therefore, their products cause less environmental pollution. On the other hand, undiversified export firms may be more constrained by limitations in capital, technology, scale, or development strategies and may meet the measures of fewer countries. Their production, processing, and shipping links may need to meet lower standards. Therefore, undiversified firms cause relatively more environmental pollution and export lower-quality agri-products. These result is same to the conclusion of Levy & Dinopoulos (2016) [39] who found more stringent environmental standards or trade liberalization policies enhance per-capita real consumption, and the effects of these policies on global pollution and welfare are ambiguous. This paper get the further conclusiton from the heterogeneous SPS measures.

### Heterogeneity across firm different trade mode

Table 7 reports the trade mode distribution of different diversified firms in China’s agri-product exports to the U.S. In processing firms and general firms, the contribution of undiversified firms is lower than that of diversified firms, and the contribution of diversified processors is the highest. Therefore, another question arises: What is the export choice of firms with different trade modes? Table 8 shows the regression results for general and processing firms. The analysis shows that: (1) the quality upgrading effect of SPS measures on undiversified firms is significantly greater than that on diversified firms, and (2) compared with processing firms, SPS measures promote general firms more. The quality-upgrading coefficient of undiversified general firms (0.066) is 2.4% higher than that of undiversified processing firms (0.042). Generally, firms are based mainly on primary products with complementary resources, have closer industrial linkages, and their degree of path dependence is stronger [38]. Meanwhile, processing firms mainly produce finished food with lower export switching costs and more target markets, which distort the positive effects of SPS measures.

**Table 7:** The export proportion of different trade mode firms in 2015 (Unit: %).

Trade mode	Undiversified export firms		Diversified export firms	
	The proportion in undiversified export value (%)	The proportion in total export value (%)	The proportion in diversified export value (%)	The proportion in total export value (%)
**General trade**	12.66	1.54	87.34	10.63
**Processing trade**	23.92	21.01	76.08	66.82

Source: estimated by the authors using GACC.

**Table 8:** The regression results of different trade mode firms.

	Undiversified export firms		Diversified export firms	
	General trade	Processing trade	General trade	Processing trade
**SPS**	0.066**	0.042**	0.040***	0.009
	(0.024)	(0.013)	(0.007)	(0.005)
**FD**	-0.075***	-0.059***	-0.045***	-0.074***
	(0.011)	(0.006)	(0.010)	(0.006)
SPS*FD	-0.005	-0.005**	-0.006***	-0.003***
	(0.003)	(0.002)	(0.001)	(0.001)
**Adjusted**_**price**_	-0.199***	-0.115***	-0.170***	-0.146***
	(0.026)	(0.011)	(0.024)	(0.015)
**Tariff**	-0.001	0.002	0.017***	0.028***
	(0.005)	(0.002)	(0.004)	(0.003)
**Cons**	0.502***	0.474***	0.286***	0.451***
	(0.070)	(0.038)	(0.069)	(0.040)
**Obs**	3278	8204	1612	4543

NOTE: values in brackets are standard deviations. ***, ** and * respectively mean statistical significance of 1%, 5% and 10%. All the models were estimated using Tobit.

From the perspective of environmental protection, in terms of the trading mode under the effect of SPS measures, the product quality upgrading of general firms is higher than that of processing firms, regardless of whether they are diversified. This also reflects the fact that environmental factors, as regulatory variables, are affected by heterogeneous SPS measures. (1) Processing firms take advantage of China’s “demographic dividend” and mainly produce labor-intensive products. They import intermediate products and then re-export the finished products, making them highly dependent on the quality of intermediate products. Therefore, the product quality is less affected by domestic water, soil, air, and other environmental factors. (2) In contrast, general firms mainly produce land-intensive products, and their product quality depends heavily on domestic water, soil, air, and other environmental factors. Therefore, in the face of international heterogeneous SPS measures, the product quality improvement scope of China’s general firms is relatively higher than that of processing trade firms, especially diversified general firms (10.63%), which impedes environmental protection when they switch to destinations with lower SPS measures.

### The effects of protective SPS standards

As discussed in 6.1 and 6.2, under the coercive effect of SPS measures, firms with different ownership types will increase their R&D investment and reduce the use of chemical elements such as pesticides and fertilizers, which optimizes the quality of water, soil, air, and other environmental factors. In addition, firms with different trading modes have different degrees of dependence on the quality of water, soil, air, and other environmental factors, and the upgrading range of the agri-product quality of general trading firms is higher.

However, SPS measures are quarantine and biosecurity measures applied to protect human, animal, or plant life or health from risks arising from the introduction, establishment, and spread of pests and diseases, and from risks arising from additives, toxins, and contaminants in food and feed. Under WTO regulations, any country can implement SPS measures that exceed international CAC measures. Therefore, SPS measures are not only international heterogeneity measures but also technical barriers to trade in implementing double measures for domestic and importing firms.

As for whether SPS measures are also applicable to domestic firms or not, based on the classification of the UNCTAD Trains database, SPS measures can be divided into three types (e.g., “yes,” “no” and “undefined”). We define SPS measures as protective measures if they are not’ treated in the same way as domestic and foreign firms. Table 9 reports the effects of protective SPS measures on the quality level and quality improvement of undiversified and diversified firms separately. The analysis shows that protective SPS measures have a significant positive effect on quality level, but no significant positive effect on quality upgrading. However, diversified firms are encouraged to maintain a higher quality level under the effect of protective SPS measures (0.061) than undiversified firms (0.035). This suggests that a multilateral agricultural cooperation, as a horizontal dimension of diversified export paths, can effectively mitigate the negative effects of trade protection by broadening export channels. Protective SPS measures impede environmental protection in China

**Table 9:** The regression results of protective SPS standards.

	Quality level		
	All	Undiversified export firms	Diversified export firms
**SPS**	0.053***	0.035*	0.061***
	-0.009	-0.017	-0.011
**FD**	0.994***	0.990***	0.995***
	-0.001	-0.002	-0.001
SPS*FD	-0.005***	-0.002	-0.006***
	-0.001	-0.003	-0.002
**Adjusted**_**price**_	-0.006***	-0.012***	-0.006***
	-0.001	-0.003	-0.001
**Tariff**	0	-0.001	0
	0	-0.001	0
**Cons**	0.019**	0.050***	0.008
	-0.006	-0.012	-0.008
**Obs**	32699	10424	22275
	**Quality upgrading**		
	**All**	**Undiversified export firms**	**Diversified export firms**
**SPS**	0.1	0.078	0.118
	-0.084	-0.164	-0.097
**FD**	-0.020**	-0.035*	-0.015
	-0.008	-0.015	-0.009
SPS*FD	-0.042***	-0.04	-0.043**
	-0.012	-0.025	-0.014
**Adjusted**_**price**_	-0.132***	-0.175***	-0.113***
	-0.02	-0.037	-0.023
**Tariff**	0.018***	0.009	0.022***
	-0.004	-0.007	-0.004
**Cons**	-1.843***	-1.852***	-1.824***
	-0.072	-0.134	-0.085
**Obs**	32699	10424	22275

NOTE: values in brackets are standard deviations. ***, ** and * respectively mean statistical significance of 1%, 5% and 10%. All the models were estimated using Tobit.

## Conclusion

This study explores the effect of SPS measures on the quality upgrading of agri-products and environmental protection from the perspective of heterogeneous international SPS measures. Based on export quality and heterogeneous SPS measures, we obtain hypothetical propositions and use the Tobit regression method to conduct empirical tests. The results show that:

(1) Affected by the effects of “path dependence,” “export switch” and “learning-by-doing,” heterogeneous SPS measures force different diversified firms to upgrade differently, which is not conducive to environmental protection in the long run. (2) Influenced by the interaction effect of the diversification degree and front distance, undiversified laggards showed the fastest upgrading, while diversified leaders showed the slowest upgrading. All the results passed the robustness test. (3) The quality upgrading made by diversified SOEs is higher than that of foreign and private firms, and the quality upgrading made by general firms is higher than that of processing firms. Besides, protective SPS measures have a stronger negative effect on quality upgrading and environmental protection than general SPS measures.

## Supporting information

S1 Appendix (DOCX)
