# Supplementary material for: Impact of sanitary and phytosanitary measures on agri-products quality upgrading and environmental protection
Source: PLoS One. 2024 Apr 5;19(4):e0297787. doi: 10.1371/journal.pone.0297787 (PMC10997108; doi:10.1371/journal.pone.0297787)
Supplement: S1 Appendix — (DOCX) [file pone.0297787.s001.docx]

Supporting information

**S1 Appendix: measures of the quality following Khandelwal et al., (2013)**

Khandelwal et al., (2013) uses the DSM quality measurement model. The premise is that when the product prices are the same, a product with a higher market share represents a higher quality. Assume that the consumer's CES utility function is:

$U=\left[ \int_{h\in H} \left[ \varphi(h)q(h) \right]^{(\sigma-1)/\sigma}d_{h} \right]^{\sigma/(\sigma-1)}$ (1)

In the formula (1), q(h) and φ(h) denote the quantity and quality of h, respectively, and σ represents the elasticity of substitution among different varieties of products in the same category. The budget constraint function for consumers in country c to purchase product h is as follows:

$q_{\mathrm{ocht}}=\left( \varphi_{\mathrm{ocht}} \right)^{\sigma-1}{(p_{\mathrm{ocht}})}^{-\sigma}P_{\mathrm{ct}}^{-1}Y_{\mathrm{ct}}$ (2)

In the formula (2),$q_{\mathrm{ocht}}$,$p_{\mathrm{ocht}}$, and $\varphi_{\mathrm{ocht}}$ represent the quantity, price, and quality level of the products of country c, consumer n, and country h of product t in period t; $P_{\mathrm{ct}}$ represents the price index of importing country at period t. $Y_{\mathrm{ct}}$ represents the income level of importing country in period t. Take the natural logarithm to the formula (2), we can sort it out and get the following regression equation, which is the core formula for measuring quality:

$\ln q_{\mathrm{ocht}}+\sigma lnp_{\mathrm{ocht}}=\alpha_{h}+\alpha_{\mathrm{ct}}+e_{\mathrm{ocht}}$ (3)

In the formula (3),$\alpha_{h}$indicates the fixed effect of the product, which can effectively control the impact of product-level variables on product quality. $\alpha_{\mathrm{ct}}$ indicates the country-time two-dimensional virtual variable. It can effectively control variables (e.g., the gross domestic product of exporting countries) that change simultaneously with the exporting country and time. $e_{\mathrm{ocht}}$ represents a residual term that includes product quality. We can get $e_{\mathrm{ocht}}$ at each product level through regression. The calculation formula of quality is as follows:

$\mathrm{quality}_{\mathrm{ocht}}=\ln\left( \hat{\varphi}_{\mathrm{ocht}} \right)=\frac{\hat{e}_{\mathrm{ocht}}}{\hat{\sigma}-1}$ (4)

In order to facilitate the comparison of the quality in different countries and obtain the overall quality level, it is inevitable to standardize the quality as follows:

$\hat{\mathrm{quality}}=\frac{\mathrm{quality}_{\mathrm{ocht}}-\mathrm{quality}_{\min}}{\mathrm{quality}_{\max}-\mathrm{quality}_{\min}}$ (5)

$\mathrm{quality}_{\min}$and $\mathrm{quality}_{\max}$ respectively represent the minimum and maximum quality level of all agricultural exporting countries in a certain year for the HS6 digital level.$\hat{\mathrm{quality}}\in$[0,1]. The quality on HS10 code can be calculated to the firm-HS8code or the nation-HS2code level.
